# Lack of Effect of Sleep Apnea on Oxidative Stress in Obstructive Sleep Apnea Syndrome (OSAS) Patients

**DOI:** 10.1371/journal.pone.0039172

**Published:** 2012-06-25

**Authors:** M. Simiakakis, F. Kapsimalis, E. Chaligiannis, S. Loukides, N. Sitaras, M. Alchanatis

**Affiliations:** 1 1st Department of Respiratory Medicine, Medical School, University of Athens, Sotiria Hospital for Chest Diseases, Athens, Greece; 2 Department of Pharmacology, Medical School, University of Athens, Athens, Greece; 3 2nd Department of Respiratory Medicine, Medical School, University of Athens, Athens, Greece; Vanderbilt University Medical Center, United States of America

## Abstract

**Purpose:**

The aim of this study was to evaluate markers of systemic oxidative stress and antioxidant capacity in subjects with and without OSAS in order to investigate the most important factors that determine the oxidant–antioxidant status.

**Methods:**

A total of 66 subjects referred to our Sleep laboratory were examined by full polysomnography. Oxidative stress and antioxidant activity were assessed by measurement of the derivatives of reactive oxygen metabolites (d-ROMs) and the biological antioxidant capacity (BAP) in blood samples taken in the morning after the sleep study. Known risk factors for oxidative stress, such as age, sex, obesity, smoking, hypelipidemia, and hypertension, were investigated as possible confounding factors.

**Results:**

42 patients with OSAS (Apnea-Hypopnea index >15 events/hour) were compared with 24 controls (AHI<5). The levels of d-ROMS were significantly higher (p = 0.005) in the control group but the levels of antioxidant capacity were significantly lower (p = 0.004) in OSAS patients. The most important factors predicting the variance of oxidative stress were obesity, smoking habit, and sex. Parameters of sleep apnea severity were not associated with oxidative stress. Minimal oxygen desaturation and smoking habit were the most important predicting factors of BAP levels.

**Conclusion:**

Obesity, smoking, and sex are the most important determinants of oxidative stress in OSAS subjects. Sleep apnea might enhance oxidative stress by the reduction of antioxidant capacity of blood due to nocturnal hypoxia.

## Introduction

Obstructive sleep apnea syndrome (OSAS) is an increasing major health concern affecting at least up to 5% of middle aged subjects in the general population. The main pathophysiologic feature in OSAS is the repetitive nature of partial or complete collapse of the upper airway leading to oxyhemoglobin desaturation of various severity. The recognition of the relation of OSAS to increased cardiovascular morbidity and mortality has increased the burden of research in the field of this relationship [1].

Oxidative stress has been considered as a major pathogenic mechanism of atherosclerosis and cardiovascular disease [2]. It represents an imbalance between the production of free radicals such as reactive oxygen species (ROS) and the antioxidant system activity that counteracts their harmful actions. ROS such as superoxide anion (O2-), hydroxyl radical (OH-) and peroxynitrite are highly reactive molecules that interact with nucleic acids, lipids, and proteins leading to injury of the cells. The presence of systemic oxidative stress in patients with sleep apnea has been investigated in the last decade as an important mechanism that link OSAS with endothelial damage and increased risk for cardiovascular consequences [2]. The cyclical hypoxia - reoxygenation sequence that characterise the obstructive events represents the major pathway related to enhanced production of free radicals similarly to the ischemia-reperfusion injury in coronary artery disease through several molecular mechanisms. Intermittent hypoxia leads to mitochondrial dysfunction, activation of enzymes that utilize oxygen such as xanthine oxidase or NADPH, leukocyte activation and endothelial cell dysfunction thus producing generation of oxidizing agents and an upregulation of inflammatory pathways [3]–[5]. Tissue hypoxia also has been implicated in transient depletion of cellular reductants resulting in disruption of the oxidation-reduction state and thus contributing to the generation of oxidative stress [6].

Several studies have indicated the presence of higher levels of oxidative stress [7]–[12] or decreased activity of the antioxidant system [13]–[16] in OSAS patients in comparison to non-OSAS controls. However there are also studies with contrary results that failed to demonstrate increased oxidative stress in OSAS [17]–[20]. This inconsistency could be explained by the influence of confounding factors such as diabetes, hypertension and mainly obesity which is a major source of oxidative stress independent of OSAS [5]. Obesity is the most common comorbidity of OSAS presenting in more than 50% of OSAS patients [1]. Obesity itself has been also considered as a chronic inflammatory situation related to cardiovascular morbidity and oxidative stress [4], [5]. Thus, it might represent the most important confounding factor in the relationship of sleep apnea with cardiovascular morbidity. In a large community cohort study, it was demonstrated that BMI along with smoking and diabetes were the most significant and independent factors associated with systemic oxidative stress markers although the contribution of coexisting sleep apnea could not be excluded [21]. The sources of oxidative stress in obesity [22] are similar to those in OSAS thus the independent role of sleep apnea in producing oxidative stress has not been fully clarified. In this study, we evaluated markers of systemic oxidative stress and antioxidant capacity in subjects with and without OSAS in order to investigate the most important factors that determine the oxidant-antioxidant status.

## Materials and Methods

### Ethics Statement

A written informed consent was obtained from all subjects and the study was approved by the Sotiria Hospital for Chest Diseases ethics committee.

### Subjects

We examined sixty-six consecutive subjects recruited from September 2007 to July 2008 from those referred for evaluation for the presence of sleep apnea to the Sleep Laboratory of a university hospital. All subjects were given a detailed medical and sleep interview and had complete physical examination and blood tests. Exclusion criteria included the presence of diabetes or any other endocrinologic diseases, renal diseases, cancer history and chronic airway disease. Patients with rhinitis, sinusitis, respiratory infections or systemic infections were also excluded. All subjects had received no therapy with inhaled, oral or nasal steroids or other anti-inflammatory drugs for 3 months prior to entry in the study. After completing overnight polysomnography we classified subjects either as controls with an Apnoea-Hypopnoea Index (AHI) ≤5 events/h or as OSAS patients with an AHI ≥15 events/h.

### Polysomnography

All subjects underwent full night comprehensive polysomnography. Monitoring included airflow via a nasal pressure transducer associated with thermistor signals, arterial oxygen saturation by pulse oximetry, respiratory efforts by abdominal and thoracic bands and electrocardiography. Sleep staging was performed via signals of electroencephalogram (EEG), electroocculogram (EOG) and submental and pretibial electromyography (EMG). An apnea was defined by a >90% decrease in the thermistor signal of airflow for ≥10 sec. An hypopnoea was defined by a >50% reduction of nasal pressure signal lasting for ≥10 sec associated with a decrease of ≥3% in oxygen saturation or an EEG arousal according to the American Academy of Sleep Medicine new scoring rules [23]. An event was defined as obstructive in the presence of respiratory efforts and central in the absence. The apnea hypopnea index (AHI) was defined as the number of obstructive and central apneas and hypopneas per hour of sleep and calculated by dividing the total number of events by the total sleep time.

### Measurements

A venous blood sample of ten milliliters was taken in all subjects at waking time (8∶00am) after overnight fasting. Five milliliters were used for determining the lipid profile (total cholesterol, LDL and HDL cholesterol and triglycerides), glucose and creatinine by routine laboratory techniques. The rest 5 milliliters were centrifuged at 6000/ min for 5 min and the serum was stored immediately at −70^C^ for measurement of oxidative stress and antioxidant capacity.

### Reactive Oxygen Metabolites (d-ROMs Test)

ROMs were measured in serum samples by the Diacron reactive oxygen metabolites (d-ROM) test (Diacron SPF, Grosseto, Italy). This test is based on the reaction of hydroperoxides of a biological sample with transition metals (iron) that catalyze the formation of free radicals which then oxidize an alchilamine forming a colored radical detected by photometry at 505 nm. Ten µL of blood are mixed with 1 ml of an acidic (PH 4.8) buffer reagent (R2 ) in order to release iron from plasma proteins that will react with peroxides of the blood to form free radicals, and then 10 µL of a chromogen reagent (R1 reagent, alchilamine ) are added forming a pink-colored derivative. This colored derivative is photometrically quantified and the optical density is directly proportional to the concentration of ROMs. Reference values of d-ROMs test are between 250 and 300 Carratelli Units (Carr. U.) i.e. 20–24 mg H_2_O_2_/dl, independently of gender and age. Values higher than 320 indicate increasing levels of oxidative stress in a linear way up to 500 carr.

### Biological Antioxidant Potential (BAP Test)

The total antioxidant capacity was measured by the biological antioxidant potential (BAP) test (Diacron SPF, Grosseto, Italy). This method evaluates the reducing power of a biological sample. A salt of trivalent iron FeCl_3_ is solved in a given colorless solution with chelation acid derivative turning its color to red due to the action of ferric ions. This solution is then decolorized by the addition of blood serum due to the reduction of ferric ions to bivalent ions (Fe^2+^) caused by the action of antioxidants. The antioxidant potential of blood plasma can be then evaluated by assessing photometrically the degree of decolorization which reflects the amount of reduced ferric ions i.e. the reducing capacity of blood.

In the BAP method, 10 µL of the blood sample is dissolved in a colored solution that has been previously obtained by mixing a source of ferric ions (FeCl_3_, ferric chloride, R2 reagent) with a special chromogenic substrate (a thiocyanate-derived compound, R1 reagent). After a short incubation (5 min), such solution will decolorize and the intensity of this change will be directly proportional to the ability of plasma to reduce ferric ions. The results are expressed in units of µmol/L. Normal reference values of BAP test are >2200 µmol/L. Values below 2000 µmol/L indicate an antioxidant deficiency status.

### Statistical Methods

Data are expressed as the mean ± SD. Spearman rank correlations test was used to explore the correlations between levels of oxidative stress (d- ROMS values) and antioxidant capacity (BAP values) with age, BMI, sleep disordered breathing (SDB) variables and lipid profile parameters. Comparisons between groups were performed using the student t-test for continuous variables and chi-square test for categorical variables. Stepwise multiple regression analyses were performed to investigate the most significant factors in predicting levels of d-ROMS and BAP. Statistical significance was defined as a p-value <0.05. All statistical analyses were made by using the software programme Statistica version.6.

## Results

### Comparison between OSAS and Non-OSAS Groups

42 patients with OSAS (mean AHI: 36.9±24.1 events/hour) were compared with 24 non-OSAS subjects (mean AHI: 3.2±2.5). In the OSAS group, patients were older than controls (49.7±12.7 vs. 42.8±13.1 years, p = 0.04) but they had similar BMI (34.4±8.5 vs. 33.3±9.5 Kg/m^2^, p = 0.62). There were no significant differences between the two groups regarding presence of arterial hypertension but the control group included a significantly higher proportion of female subjects and smokers (see table 1). There was no significant difference in the lipid profile between the two groups.

**Table 1:** Comparison between OSAS patients and control subjects.

Variable	OSAS (n = 42)	Controls (n = 24)	p-value
Sex (M:F)	27∶15	12∶12	
Smokers (n-%)	16 (38%)	18(75%)	<0.01
Hypertension (n-%)	12 (28%)	6 (25%)	0.45
Age (yrs)	49.7±12.8	42.9±13.1	0.04
BMI (Kg/m^2^)	34.4±8.5	33.3±9.5	0.62
AHI(events/h)	36.9±24.1	3.2±2.5	<0.001
Nadir SaO_2_(%)	78.6±9.7	90.9±2.5	<0.001
TST<90% (%)	16.7±22.7	0.08±0.4	<0.001
d-ROMs (Carr.U)	404.3±98.3	476.4±98.9	0.005
BAP (µmol/L)	2057.2±561.7	2410.3±304.9	0.005
Cholesterol (mg/dl)	224.1±37.1	209.2±39.5	0.13
LDL(mg/dl)	150.5±30.9	132.5±37.5	0.04
HDL (mg/dl)	47.9±10.4	52.3±13.1	0.14
Triglycerides (mg/dl)	139.7±73.0	116.1±65.7	0.19

The mean level of d-ROMs was significantly higher in the control group (404.3±98.3 vs. 476.4±98.8, p = 0.005) but the mean value of antioxidant capacity as evaluated by BAP was significantly lower in the OSAS group (2092.9±467.6 vs. 2410.4±304.9, p = 0.004). Only 8% of the controls compared with 21% of patients had values of d-ROMS in the normal range (below 320). In contrast, as regarding the BAP values, 52% of patients had values <2200 compared with only 4% (1 subject) in the control group.

### Correlations

In the whole sample of participants we found that the serum levels of oxidative stress as expressed by D-ROMs levels had a significantly positive correlation (see table 2) only with the BMI (r = 0.332, p = 0.006) and high density lipoproteins (r = 0.266, p = 0.03). There was also a significant negative correlation with the AHI (r = −0.259, p = 0.03) and no correlation with the nadir SaO_2_ (r = 0.209, p = 0.09) and total sleep time with SaO_2_<90% (r = −0.222, p = 0.18). Instead, the levels of antioxidant capacity as evaluated by the BAP test (see table 3) were positively correlated with the nadir SaO_2_ (r = 0.294, p = 0.01) and negatively correlated with triglycerides levels (r = −0.287, p = 0.01), cholesterol (r = −0.268, p = 0.03) and low density lipoproteins (r = −0.287, p = 0.01). There was no significant correlation between the BAP values and the AHI (r = −0.191, p = 0.12), the total sleep time with SaO_2_<90% (r = −0.167, p = 0.17) or the BMI (r = −0.050, p = 0.68).

**Table 2:** Correlations of d-ROMs metabolites.

Variable	Spearman r	p-value
Age	−0.121	0.33
BMI	0.332	0.006
AHI	−0.259	0.03
TST<90%	−0.222	0.18
Nadir SaO_2_	0.209	0.09
Cholesterol	−0.197	0.11
HDL	0.266	0.03
LDL	−0.226	0.06
Triglycerides	−0.124	0.31

**Table 3:** Correlations of BAP.

Variable	Spearman r	P value
Age	−0.012	0.92
BMI	−0.050	0.68
AHI	−0.191	0.12
TST<90%	−0.167	0.17
Nadir SaO_2_	0.294	0.01
Cholesterol	−0.268	0.03
HDL	0.162	0.19
LDL	−0.287	0.01
Triglycerides	−0.386	0.001

### Multiple Regression Analyses

Separate multiple regression analyses were done with levels of d-ROMs and BAP values as the dependent variables in order to examine the most significant factors that could explain their variance. Independent variables included BMI, age, smoking habit, presence of hypertension, AHI, nadir SaO_2_, TST<90%, total cholesterol, HDL, LDL and triglycerides. As regarding the level of oxidative stress as expressed by the d-ROMs levels, in a model explaining about 32% (adjusted R^2^ = 0.32) of variance adjusted for confounding factors such as sex and age, the most significant predicting factors in the model were the BMI (beta = 0. 298, p = 0.02) and smoking habit (beta  = 0.229, p = 0.03) and sex (beta = 0.440, p<0.001). Parameters of SDB such as the AHI, nadir SaO_2_ and TST<90% or parameters of lipid profile were not significant predicting variables. As regarding the level of antioxidant capacity (BAP), minimal oxygen desaturation (beta = −0.240, p = 0.02), and smoking habit (beta = −0.150, p = 0.04) were the most significant factors included in a model that explained about 25% of the variance. The AHI and TST<90% or the BMI were not included in the model.

## Discussion

Our results indicate that systemic oxidative stress in patients with OSAS is not associated with the severity of sleep apnea itself but rather with the presence of obesity and smoking habit. The contribution of sleep disordered breathing in the imbalance of oxidant-antioxidant status might be better explained with a reduction of the antioxidant capacity in the presence of significant hypoxia.

Oxidative stress has been assessed by direct measurements of ROS and enzymes which are involved in ROS production or indirect measurements of oxidized products of lipids, proteins, or DNA. Reactive oxygen species (ROS) and free radicals have a very short life making their direct measurement difficult. In this study we used a simple method of evaluating the systemic oxidative stress that has been developed in order to measure the concentration of hydroperoxide induced by free radicals [24]. It has been considered to be directly proportional to the quantity of reactive oxygen metabolites (ROMs) indicating the level of oxidative stress throughout the body.

Several studies have suggested the presence of increased oxidative stress in sleep disordered breathing using various markers of oxidative stress but there are many studies with contrary findings. Earlier investigations [7], [8] demonstrated increased production of ROS in leucocytes of OSAS patients when compared to controls. Other markers such as increased lipid peroxidation [9], [10], [14] and oxidized DNA [11] assessed by oxidized LDL autoantibodies, thiobarbituric acid reactive derivatives (TBAR) and urinary 8-hydroxy-2′-deoxyguanosine excretion have been found higher in subjects with OSAS. In contrast, other studies [17]–[20] could not demonstrate increased oxidative stress in OSAS patients when compared with controls. Thus whether there is increased oxidative stress in OSAS remains controversial.

Oxidative stress has been related to many factors that could be acting as confounding factors in the relationship with sleep apnea.These include obesity, smoking, age, hypertension, hyperlipidemia and diabetes [21]. In our study we investigated all these possible confounding factors except for diabetes. We found that obesity and smoking habit had a greater influence on the development of oxidative stress than sleep apnea. The mean levels of d- ROMs, which represent an overall estimation of oxidative stress although found to be abnormally high in sleep apnea patients were not higher than those in controls with similar BMI. In fact, level of oxidative stress was found lower in sleep apnea subjects despite similar degree of obesity, a finding that could be explained by the higher percentage of smokers and females in the control group. In multiple regression analysis, oxidative stress was mostly associated with obesity, smoking habit and female sex. Thus, although we found increased oxidative stress in OSAS, it was better explained by the influence of obesity, smoking and sex.

Our findings might be in contrast with previous studies showing increased oxidative stress in OSAS but the confounding role of obesity has not been well elucidated. In a recent study, the authors reported higher TBAR in patients when compared obese patients with obese controls but they failed to find higher oxidative stress in overweight patients in comparison to overweight controls [14].Other studies showed increased lipid peroxidation [9] or levels of oxidized DNA [11] in OSAS patients in comparison with controls but the control groups had a significantly lower BMI. Enhanced levels of d-ROMS [12] have been reported in OSAS in comparison with controls of similar BMI but the mean values of d-ROMS were not different between the two groups indicating that high levels of d-ROMs in patients could be explained by the presence of obesity. Significant differences between patients and controls have been found only when obese patients are compared with non-obese controls with attenuation of the difference [18] when obese patients are compared with obese controls.

According to recent evidence, obesity is an inflammatory state linked to increased oxidative stress [22]. Several studies in community based samples showed that BMI was independently associated with systemic oxidative stress markers although the presence of SDB has not been into consideration in general population studies [21]. The mechanisms that link obesity with oxidative stress are similar to those have been investigated in OSAS [4], [5]. It has been proposed that oxidative stress in obesity is a response to hypoxia because of increased distance of the enlarged adipocytes from the vasculature [25]. The common coexistence of obesity with OSAS [1] and the common pathways to oxidative stress and inflammation might explain the confounding role of obesity in the relationship of sleep apnea with increased oxidative stress.

The antioxidant status is the other counterpart in the generation of oxidative stress. Since obesity, smoking or metabolic dysregulation which are the most important factors leading to oxidative stress present commonly in patients with OSAS the role of sleep apnea could be explained by the enhancement of the oxidative stress that already exists. The reduction of antioxidant activity by sleep apnea could result in an imbalance of oxidant-antioxidant status in favour of oxidative stress.

Several studies [13]–[16] have shown that obese or non-obese sleep apnea patients have decreased antioxidant capacity in comparison with controls. In contrast, other authors [17], [19] have found no difference in antioxidant enzymes activity between OSAS and controls. In this study, we examined the antioxidant activity with a test (BAP) that evaluates the total antioxidant capacity of the blood. This test has never been investigated in OSAS patients but it has been considered as a method with reproducible results and used as a marker of antioxidant activity in cardiovascular surgery [26]. We found that OSAS patients had decreased values of BAP in comparison with control subjects. Smoking is a known condition that reduces antioxidant capacity and produces oxidative stress [21], [27]. Although our controls included more smokers than OSAS patients they had higher values of BAP. However, the independent role of sleep apnea was indicated by the significant predicting value of minimal saturation. However, the AHI was not an important predicting factor of antioxidant status in contrast to previous studies. Thus our results demonstrate that hypoxia may have an additional effect on antioxidant activity independent of obesity or smoking in agreement with previous studies showing that antioxidant capacity is reduced in nonobese patients [15] or overweight and obese patients [14]. Mechanisms of hypoxia induced reduction of antioxidant capacity may be associated with dysregulation of genes involved in the modulation of ROS or enzymes involved in the production of the antioxidant barriers.

There are several potential limitations to our study. The nature of this study is mostly descriptive and causal relationships cannot be detected. The number of OSAS (i.e. 42) patients was not equal to the number of control subjects (i.e.24) because it was difficult to have many subjects with obesity and no evidence of OSAS. Moreover, our control group included subjects referred with symptoms of sleep apnea and not healthy individuals. Thus there is not a control group of nonobese, nonsmokers individuals that would elicitate the relationship of oxidative stress with indices of OSAS. For this reason, we used a current definition of hypopnea that detects even minor degrees of SDB including upper airway resistance in order to avoid the inclusion of mild SDB in the control group. We did not exclude smokers and women from our study population. This could have considerable bias effect on our results since female sex and smoking are strongly associated with enhanced oxidative stress [27]. However this did not weaken our results since although more smokers and women were included in the control group the significance of nocturnal hypoxemia was effectively detected. The presence of more female subjects in the control group could explain the higher values of oxidative stress in the control group. The lack of data on the menopause status is also a limitation of this study since presence of menopause could have an effect on oxidative stress. In our nocturnal polysomnograms, we did not routinely monitor end tidal carbon dioxide, thereby we are unable to determine the possibility of a confounding influence of obesity hypoventilation in some obese patients that could account for increased oxidative stress. Finally, our methods for estimating oxidative stress and antioxidant capacity reflect the overall status and excludes non-hydroperoxide related ROS mediated markers and also cellular anti-oxidant capacity (glutathione, mitochondrial).

In conclusion, our results showed that obesity, smoking and sex are the most important determinants of oxidative stress in OSAS subjects. Sleep apnea might enhance oxidative stress through a reduction of antioxidant capacity of blood due to hypoxia related to respiratory events. Certainly prospective studies separately in men and women with more and preferably non-obese and non-smoking subjects are needed to clarify the independent role of OSAS in producing oxidative stress.
